# Minimum Pricing of Alcohol versus Volumetric Taxation: Which Policy Will Reduce Heavy Consumption without Adversely Affecting Light and Moderate Consumers?

**DOI:** 10.1371/journal.pone.0080936

**Published:** 2014-01-22

**Authors:** Anurag Sharma, Brian Vandenberg, Bruce Hollingsworth

**Affiliations:** 1 Centre for Health Economics, Monash University, Melbourne, Australia; 2 Cancer Council Victoria, Melbourne, Australia; 3 Division of Health Research, Lancaster University, United Kingdom; CUNY, United States of America

## Abstract

**Background:**

We estimate the effect on light, moderate and heavy consumers of alcohol from implementing a minimum unit price for alcohol (MUP) compared with a uniform volumetric tax.

**Methods:**

We analyse scanner data from a panel survey of demographically representative households (n = 885) collected over a one-year period (24 Jan 2010–22 Jan 2011) in the state of Victoria, Australia, which includes detailed records of each household's off-trade alcohol purchasing.

**Findings:**

The heaviest consumers (3% of the sample) currently purchase 20% of the total litres of alcohol (LALs), are more likely to purchase cask wine and full strength beer, and pay significantly less on average per standard drink compared to the lightest consumers (A$1.31 [95% CI 1.20–1.41] compared to $2.21 [95% CI 2.10–2.31]). Applying a MUP of A$1 per standard drink has a greater effect on reducing the mean annual volume of alcohol purchased by the heaviest consumers of wine (15.78 LALs [95% CI 14.86–16.69]) and beer (1.85 LALs [95% CI 1.64–2.05]) compared to a uniform volumetric tax (9.56 LALs [95% CI 9.10–10.01] and 0.49 LALs [95% CI 0.46–0.41], respectively). A MUP results in smaller increases in the annual cost for the heaviest consumers of wine ($393.60 [95% CI 374.19–413.00]) and beer ($108.26 [95% CI 94.76–121.75]), compared to a uniform volumetric tax ($552.46 [95% CI 530.55–574.36] and $163.92 [95% CI 152.79–175.03], respectively). Both a MUP and uniform volumetric tax have little effect on changing the annual cost of wine and beer for light and moderate consumers, and likewise little effect upon their purchasing.

**Conclusions:**

While both a MUP and a uniform volumetric tax have potential to reduce heavy consumption of wine and beer without adversely affecting light and moderate consumers, a MUP offers the potential to achieve greater reductions in heavy consumption at a lower overall annual cost to consumers.

## Introduction

Alcohol consumption is among the top three risk factors for global disease burden, accounting for 5.5% of disability-adjusted life years lost, behind tobacco smoking including second-hand smoke (6.3%) and high blood pressure (7.0%) [1]. Increasing the cost of alcohol to consumers, through government pricing and taxation policies, has been shown to be effective in reducing overall consumption in the population, rates of heavy drinking, and the incidence of alcohol-related harm, whether it is implemented with other complementary alcohol strategies, or on its own [2], [3]. Evidence reviews suggest that a public health-orientated alcohol pricing and taxation system is one that (i) increases the minimum price at which alcohol can be purchased, and/or (ii) taxes products on a volumetric basis (i.e. according to alcohol content), with the aim of deterring initiation into drinking and recognising that among current drinkers it is the volume of alcohol consumed on single occasions and over time that increases health risks [2], [4], [5], [6].

A motivation for our study is very recent debates, in several countries around the globe, regarding the desirability of various pricing and taxation policies for alcohol [7]. Of particular interest to us are the developments in Scotland where, in May 2012, the Parliament passed legislation to introduce a minimum unit price (MUP) for alcohol. At the time of writing, the Scottish legislation is yet to be implemented amid legal challenges from a number of European countries [8]. This highlights the fierceness of debate regarding such policies and the critical importance of building the empirical evidence base to inform decision-making in the area.

Reforms to pricing policies on alcohol are also being contested in England. During 2012, the British Prime Minister expressed support for introducing a MUP and initiated consultations with industry and health groups, receiving strong support from the latter [9]. However, in July 2013, the government reversed its position on MUP in handing down a report that the consultation process:

*“…has not provided evidence that conclusively demonstrates that Minimum Unit Pricing (MUP) will actually do what it is meant to: reduce problem drinking without penalising all those who drink responsibly. In the absence of that empirical evidence, we have decided that it would be a mistake to implement MUP at this stage”* [10].

In Australia, a recent independent panel review of the alcohol taxation system, commissioned by the government, judged it to be ‘incoherent’ and recommended major reforms so that ‘all alcoholic beverages should be taxed on a volumetric basis, which, over time, should converge to a single rate, with a low-alcohol threshold introduced for all products [11]. Such a tax is also a relatively simple policy to implement, as noted by the review: ‘a uniform rate of tax across all beverages is the least complex and most efficient way of imposing an alcohol tax [11]. The rate of this new volumetric tax, the panel argued, ‘should be based on evidence of the net marginal spill over cost of alcohol’ [11]. However, the review panel cautioned that a uniform volumetric tax may not be effective at targetting only at those drinkers most likely to cause social harm. This could mean “*consumers who enjoy alcohol responsibly (light and moderate drinkers) might face an unnecessarily high price (and pay too much tax)*” [11]. Also, importantly, the review noted that while uniform volumetric tax would provide a floor price, alcohol could still sometimes be sold below cost or given away [11].

Despite the continuing call for reform from both health and industry groups, further discussions on volumetric taxation and MUP policies have now reached an impasse in Australia, with some groups voicing strong support for the introduction of MUP, while others have called instead for the introduction of a new volumetric tax, in part to replace the *ad valorem* (valued based) tax on wine products [12]. To some extent, in this context of current policy discussion in Australia, volumetric taxation and a MUP are framed as two alternative options. We view this as a clear opportunity and motivation to examine the respective effects of each policy, and in doing so we aim to provide timely evidence to the Australian and international scientific and policy community.

### Current practices and expected effects of volumetric taxation and minimum pricing

Internationally there is a growing trend towards setting alcohol taxes on a volumetric basis [13], and there are numerous studies highlighting the effectiveness of this approach to reduce heavy drinking and alcohol related harm [14], [15], [16]. In more than 30 developed countries throughout the world alcohol is taxed either on a volumetric or *ad valorem* basis [17]. While it is uncommon for countries to adopt only one of these methods, some, such as Vietnam, have recently simplified alcohol excise duty rates that varied for different alcohol products with a new volumetric tax [13], and the trend internationally is towards setting alcohol excise rates that reflect the alcohol content of products [6]. However, the current practice in many jurisdictions is often a very complex combination of both volumetric and *ad valorem* taxes, and how each of the two methods of taxation apply often varies by the type of alcohol product (i.e. beer, wine, spirits) and by sub-categories within these types, and also by specific ranges of alcohol content. In some jurisdictions, alcohol taxes also differ between imported and locally produced products. Furthermore, public health objectives are not usually the main influence on alcohol taxation policy, with government revenue objectives as well as political and commercial interests often being the main factor. Australia is typical of the complex alcohol tax arrangements that can emerge in this context, which are summarised well by Byrnes *et al* [18]:

*Within Australia, volumetric excise taxes are levied on beer and spirits based on pure alcohol content and an ad valorem excise tax is levied on wine based on the wholesale price. In addition, there are different tax rates applied to beer depending on the total strength of alcohol and the container size (‘kegs’ containing more than 48 litres are taxed substantially less per litre alcohol than beer sold in individual containers). Furthermore, the first 1.15% alcohol content in beer is tax free, a threshold that is not provided for any other alcohol beverage. As such, there exists a large divide regarding the amount of tax charged for the equivalent amount of pure alcohol consumed. For example, low strength draught (i.e. from a keg) beer and cheap wine are taxed considerably lower than spirits for the same amount of pure alcohol.*

From a public health perspective, the advantage of volumetric taxation over *ad valorem* taxation is that the latter may lead to some producers ‘downgrading’ the quality and cost of their products, resulting in relatively low tax on some beverages despite their high alcohol content [5]. A potential disadvantage of volumetric taxation, on the other hand, is that it may result in some consumers switching to cheaper products as it does not prohibit alcohol from being heavily discounted or sold below cost [19]. To address this, a separate, or supplementary policy, which has been suggested is a MUP [20]. However, in the absence of cross-national empirical evidence on the effects of a MUP, arguments have ensued about its impact, including: that it may not actually deter heavy drinking; that it may adversely affect the majority of people who are light or moderate drinkers; and, that it may disproportionately and hence, unfairly, impact upon low-income drinkers [12], [21]. In view of this, a main aim of our study is to determine which of these two policies – a volumetric tax or a MUP, is most effective in reducing heavy consumption and what is the associated effect on light and moderate consumers.

A MUP for alcohol establishes a government regulated price for a specified volume of pure alcohol or alcoholic beverage below which products may not be sold. Depending on the value at which a MUP is set, it may not necessarily lead to large price increases in all products [see Table 1]. The appeal of a MUP from a public health perspective is that it increases the price of the cheapest alcohol products, which could potentially reduce heavy consumption and, in turn, reduce rates of alcohol-related harm [18], [20], [22], [23], [24], [25].

**Table 1:** An example of how a minimum unit price (MUP) for alcohol affects alcohol prices.

**Product characteristics**		
Product type		Beer
Product brand name		Golden Lager
Alcohol by volume, %		3.5
Unit volume, litres of beverage		0.375
Pack size, number of units		30
Pack volume, litres of beverage		11.25
**Volume purchased**		
Number of packs purchased		2
Total volume purchased, litres of beverage		22.50
Total litres of pure alcohol (LALs) purchased		0.7875
Total number of standard drinks (0.01267 LALs) purchased		62.15
**Original prices and taxes, A$**		
After-tax retail price^a^	Per standard drink:	0.77
	Per unit:	0.80
	Per pack:	24.00
	Total spend:	48.00
Original volumetric tax^b^	Per standard drink:	0.35
Original 10% Goods and Services Tax (GST) when purchased	Per standard drink:	0.07
Base price^c^	Per standard drink:	0.35
**Estimated prices after applying a MUP** d **, A$**		
Original after tax retail price	Per standard drink:	0.77
MUP inflation	Per standard drink:	0.23
New after tax retail price	Per standard drink:	1.00

^a^ Inclusive of original volumetric taxes (excise) and original 10% Goods and Services Tax (GST).

^b^ A$41.68 per LAL above 1.15% ABV when purchased.

^c^ Exclusive of all original volumetric taxes (excise) and original 10% Goods and Services Tax (GST).

^d^ Inflation of prices to $1.00 per standard drink if the original after tax retail price was <$1.00 per standard drink.

While there is increasing international interest in MUP policies, to date, very few jurisdictions have adopted this approach and, subsequently, there is limited evidence on its effects. The only demonstrated effects of a MUP on alcohol consumption at present are limited to empirical studies in the Canadian provinces of British Columbia and Saskatchewan, which found that a 10% increase in the minimum price of all beverages reduced total consumption in each province by 3.4% and 8.4%, respectively [26], [27]. Another empirical study in Canada, examining the relationship between a MUP and alcohol attributable (AA) deaths, found that 10% increase in the average minimum price for all alcoholic beverages was associated with a 32% reduction in wholly AA deaths [28]. However, none of these studies looked at changes in alcohol consumption, or AA deaths, by type of drinker. Researchers in the UK modelled the effect of various price policies on different sub-groups of drinkers and found that a MUP of £0.50 per unit of alcohol would achieve a greater reduction in overall consumption and heavy drinking than a ban on promotions of discount alcohol or increasing the current retail price of all product categories by 10% [19], [29], [30]. They also report that compared to the latter option, a MUP of £0.50 per unit would impose a smaller annual cost increase on moderate drinkers. However, comparisons with the effects of volumetric taxation options were not included in their study.

Hence, our study is very timely and, to the best of our knowledge, is the first to provide an accurate estimate and comparison of the effects on light, moderate and heavy alcohol consumers of introducing a minimum price versus a uniform volumetric tax on off-trade alcohol.

We report changes in the estimated cost (changes in mean annual consumer spending per capita on alcohol) and changes in mean annual volume of alcohol consumed per capita resulting from each of the policy options. Our main finding is that while both a minimum price and a volumetric tax have potential to reduce heavy consumption of wine and beer without adversely affecting light and moderate consumers, a minimum price offers the potential to achieve greater reductions in heavy consumption at a lower overall cost to consumers. We further show through sensitivity analysis that this finding is robust to household composition, different tax pass-through rates, and implementing these two policy options simultaneously or separately.

## Methods

### Data

Our data consists of 885 households participating in the Nielsen Company's HomeScan panel survey (also called scanner data) who recorded their off-trade alcohol purchases brought home using a barcode-scanning device. The HomeScan dataset includes a unique level of detail on individual household alcohol purchases that is not provided in other publicly available population survey datasets, such as alcohol type, brand, flavour, size (millilitres), quantity, packaging (e.g. multi-pack), price paid per item (A$), total spend per shopping trip, and the date and location (i.e. store name) of the shopping trip, along with social, demographic, economic and attitudinal information about the individual household and the shopper. All households in our dataset participated in the HomeScan panel for a 52-week period, from 24 January 2010 to 22 January 2011, and reside in the state of Victoria, which is Australia's second most populous state.

HomeScan data have been used extensively for research into consumer behaviour in relation to food and nutrition, particularly in the US [31], [32], [33], but has only been used in a small number of alcohol studies [34], [35]. Access to panel expenditure datasets such as HomeScan is rare, particularly for studying alcohol purchasing behaviour. Generally, alcohol researchers and policy makers rely upon periodic, self-report population surveys of household alcohol expenditure or drinking patterns to monitor and analyse alcohol consumption [36]. The limitations of such surveys are well documented and include sampling bias, response bias, measurement bias, and recall bias, with under-reporting of consumption by heavy drinkers seen as a common weakness [36]. Annual estimates of national per capita consumption of alcohol provide a more reliable indicator of total consumption, but in many jurisdictions these are reported at a national population level only, thus constraining their usefulness for studying consumer behaviour in detail.

The appeal of HomeScan panel data, therefore, is that it overcomes some of the limits of existing surveys by collecting information on each household's alcohol purchasing constantly over 52-weeks, and includes disaggregated detail about daily shopping trips and individual products purchased by each household. A validation study of HomeScan data in the US found that households reported single purchases 99% accurately and multiple purchases 86% accurately (when checked against stores' sales records), and the small level of recording errors is similar to other research datasets for which cross-validation studies have been undertaken [37]. However, it should be noted while off-trade alcohol represents the majority (78%) of the total drinks market in Australia [38], HomeScan data does not include all households' entire off-trade alcohol purchases, as some are likely to be not bought home and scanned. For example, a household's shopper may occasionally purchase off-trade alcohol without returning home before consuming it at another location or giving it away.

For the purposes of estimating the effect of alcohol taxation and pricing policies on consumers, alcohol sales/expenditure data such as ours is very attractive because of its relative robustness and coverage. A recent study by Robinson *et al* [39] found that data on alcohol sales records are, in general, a robust source of data for monitoring alcohol consumption. A study by Ramstedt [40] found that self-reported alcohol *purchases* achieve a higher coverage rate than found typically in studies based on self-reported *use* of alcohol. Furthermore, Ruhm and colleagues [41] observe that the most useful estimates of price elasticity of demand for alcohol are obtained using annual Uniform Product Code (UPC) barcode scanner data on grocery store alcohol prices, as this accurately provides the price of each individual alcoholic beverage product.

To ensure the panel is a representative sample of the Australian population, the HomeScan panel is built using a sampling frame based on Australian Bureau of Statistics information on the geographic, demographic, social and economic distribution of the Australian population. Recruitment to the panel is determined by the primary shoppers' attributes. Key attributes used to filter recruitment to the panel include family type (life stage), household size, age, and income. The classification based on life stage is:

- Young Singles/Couples: All Adults <35, no children, size 1 or 2;
- Young Families: Adults/Shopper Any Age, and all children <11;
- Older Families: Adults/Shopper Any Age; at least 1 person aged 11–19, but no children <11;
- Older Singles/Couples: All Adults aged 45+, no children, size 1 or 2; and,
- Adult Households: All Adults aged 21+; excludes (i) to (iv).

The distribution of households in our sample by life stage (family composition) is broadly similar to that found in the most recent Australian Census of Population and Housing [42]. For example, using the figures reported by the Census and 2010 National Drug Strategy Household Survey [43] (of individuals aged 14+ years), it is estimated that around 37% of couples with children consume alcohol. The corresponding figure from our sample is of similar magnitude (33.5%). Similarly, the median income of families in our sample is $78500, which is similar to that reported in the Census ($77000 per annum) [43].

### Estimating distribution and apparent consumption of alcohol

Alcoholic content (% alcohol by volume) and the number of standards drinks (12.67 ml of pure alcohol) were manually coded to all individual alcohol products in our dataset, as this was not included within the original HomeScan data file. We were then able to accurately calculate the litres of pure alcohol (LALs) and number of standard drinks purchased at a household and per capita level, as well as the mean prices paid per standard drink for all beverage types. For example, we have information that on 24^th^ March 2010, a brand of mid-strength beer was purchased from a supermarket, as a pack containing 30 units with 375 ml in each unit, and two packs were purchased at a price of $24.00 per pack. The quantity of beer in each pack was 11.250 litres, and the ABV for this beer is 3.5%. Using this detailed information, we first calculate the total spending on beer which is $48. We then calculate total quantity of beer in ml which is 22,500 ml and convert the total quantity into the number of standard drinks using the following formula: standard drink = (quantity*ABV)/(12.67*100). This equates to a total of 62.15 standard drinks in our example. Next, we calculate price per drink by dividing total spend $48.00 by 62.15, which, in this example, equates to 77 cents per standard drink.

Our approach to examine the distribution and levels of alcohol consumption across the sample of households involves as a first step, dividing the total per capita volume of alcohol purchased across five quantiles (quintiles) of the households in our sample using ‘xtile’ command in Stata software (Version 11). This ensures that quintiles are data determined objectively and not based on any ad-hoc cut-offs or other subjective thresholds. This approach has been used widely to examine the distribution of alcohol consumption in the population, whereby the cumulative percentage of alcohol consumed is divided across percentile groupings of the population [20], [44], [45]. We include all persons aged 12+ years within each household as the denominator, as the usual age category used internationally for such estimates (persons aged 15+ years) [36] is not disaggregated in the dataset.

### Estimating changes in cost and consumption

Here we estimate the effects of applying a MUP of A$1.00 per 12.67 mls of alcohol (i.e. 1 standard drink measure in Australia) sold in off-trade products [See Table 1]. While comparisons between countries are not straightforward, in England and Scotland the proposed MUP for the equivalent volume of alcohol is £0.57 (A$0.87) and £0.63 (A$0.96), respectively. In the Canadian provinces, where minimum prices vary widely according to product type, alcohol volume, and outlet, the MUP per 12.67 mls of alcohol ranges from CAD$0.48 (A$0.46) to CAD$2.04 (A$1.96) as at June 2010 [46]. We chose a MUP of A$1.00 per standard drink for our study as it equates closely to the proposed minimum prices in the UK. We also note an Australian report that claimed a minimum price of A$1.00 per standard drink would remove very cheap (<A$0.50) products that are the choice amongst the heaviest drinkers, would reduce alcohol consumption and related harms most amongst disadvantaged populations and young people, but would not affect the price of relatively expensive products that the majority of moderate drinkers purchase [47].

We estimate the effects of a new uniform volumetric tax on all beverages as recommended recently to the Australian government by the panel that reviewed the alcohol tax system [11] [see Table 2]. Their recommended uniform volumetric tax rate, based on Cnossen's [48] estimate of the rate required to recover the full external costs of alcohol, is equivalent to that for off-trade beer with an alcohol content >3.5% (A$41.68 per litre of alcohol (LAL)) at the time our data were collected, with an exemption from taxation for the first 1.15% of alcohol in all beverages [49]. While the effect of a MUP on after-tax retail prices is reasonably clear (i.e. prices must rise to at least the stipulated minimum), the effect of a volumetric tax on after-tax retail prices is less straightforward because there is usually no mandatory requirement for producers or retailers to adjust prices in line with tax changes so long as the required amount of tax is paid to the government. There is only a small body of empirical evidence on the extent to which alcohol taxes are passed-through to consumer prices. An alcohol tax pass-through rate is calculated by dividing the real change in price by the amount of tax increase for a beverage of specific type and size. Understanding pass-through rates from tax changes to consumer prices is key to appreciating how tax changes can affect consumers, producers, retailers and society as a whole [50]. While Australian modelling studies often assume that changes in alcohol tax are passed on to and paid in full by consumers [18], [51], [52], within the international scientific literature there is considerable heterogeneity in reported pass-through rates. Factors that can contribute to the variation in pass-through rates includes market structure, geographic location, consumers' beverage preference, brand, store type, and the status of other alcohol policies [4], [50]. Some studies report that alcohol tax increases are more than passed-through in full to consumer prices [53], [54], [55]. Conversely, some studies have found alcohol taxes are less than fully passed-through to prices, and sometimes result in a negative change in prices paid by consumers, possibly as a result of downshifting by drinkers to lesser quality products [35]. A UK government report observed that large supermarkets, which have significant purchasing power, often cross-subsidise alcohol products which can mean prices do not rise as much as tax increases, whereas pubs do not have the same options and hence prices in these outlets often rise by more than the tax increase [56]. Given the range of pass-through rates reported in the literature, and the variation in pass-through rates between products types and outlet types, we test a range of assumed pass-through rates in our sensitivity analysis, reported further below.

**Table 2:** Method for estimating the effects of a uniform volumetric tax on prices.

**Step 1**		
Using detailed information on every individual product purchased by households in our dataset, we calculate the base price for each individual product (i.e. exclusive of original excise (on beer and spirits), the original WET tax (on wine), and the original 10% Goods & Services Tax (GST)) by deducting the estimated tax component in each from the individual product's original after tax retail price.		
Examples		
Product brand name	Mountain Top Merlot	River Crossing Chardonnay
Product type	Table wine	Cask wine
Alcohol by volume (ABV) (%)	14.0	14.0
Unit volume (litres of beverage)	0.75	4.0
Litres of alcohol (LALs)	0.105	0.560
Original after tax retail price:	$10.00	$12.00
Original WET tax component	$1.45	$1.74
Original GST component	$0.91	$1.09
Base price	$7.64	$9.17
**Step 2**		
We then calculate the new amount of volumetric tax applicable to each individual product (i.e. A$41.68 per LAL, excluding the first 1.15% of alcohol in each product).		
Examples (continued from Step 1):		
LALs excluding first 1.15% ABV	0.09638	0.514
New volumetric tax	$4.02	$21.42
**Step 3**		
We add the new uniform volumetric tax amount to the base price for each product, apply the 10% GST, and then calculate new mean after tax retail prices for all product types. We also assume a range of different tax pass-through rates as part of a sensitivity analysis.		
Examples (continued from Step 2)		
Base price+new volumetric tax^a^	$11.66	$30.59
New GST	$1.17	$3.06
New after tax retail price:	$12.83	$33.65

^a^ Full pass-through rates of the new volumetric tax are assumed in this example.

### Consumer responsiveness to price changes

Systematic reviews show that while consumer demand for alcohol is price responsive, it is relatively inelastic, with an average own-price elasticity of around −0.5 [57], [58]. That is, demand for alcohol, measured by consumption, is reduced by half of one per cent for every one per cent that the price is increased. An important consideration in examining pricing and taxation policies is the heterogeneity in price elasticity among drinkers. Previous studies have shown that significant differences in price responsiveness exist between drinkers depending on their country-status [59], age [57], sex [20], socio-economic status [60], beverage preference [61], the quality of their beverage [24], and pattern of drinking (i.e. heavy drinking) [62].

Given the focus of our study is to compare price responsiveness among light, moderate and heavy consumers, and elasticity is known to vary by different categories of drinkers, we assign recently published own-price alcohol elasticities for Australian drinkers to the consumers (within households) in our sample, differentiated by drinking pattern and product type [see Table 3] [63]. For these purposes, we assume consumers in our sample to be heavy drinkers if their household's average annual per capita alcohol consumption exceeds 2 drinks/day. This is the case for those in the 4^th^ and 5^th^ quintiles of alcohol consumption in our sample [see Table 4]. Similarly, we assume consumers in the 2^nd^ and 3^rd^ quintiles to be moderate drinkers, and those in the 1^st^ quintile to be light drinkers. We are cognisant that this approach of using household per capita consumption assumes all adults in each household consume same amount of alcohol, which may not be the case. We therefore test the robustness of our methodology as part of sensitivity analysis described later.

**Table 3:** Estimated own-price elasticities for households by drinking pattern sub-group, consumption volume quintile, and product type.

	Drinking pattern (Consumption volume)		
Product type	Light (1st quintile)	Moderate (2nd and 3rd quintiles)	Heavy (4th and 5th quintiles)
Wine	−0.53	−0.42	−0.28
Beer	−0.49	−0.39	−0.26
Spirits	−1.28	−1.01	−0.68
RTDs	−0.89	−0.70	−0.47

Source: Adapted from MJA 2012 [63].

**Table 4:** Summary of key alcohol purchase variables by consumption quintiles of households.

Consumption volume^a^	Households in sample (% of total)	Mean annual volume of alcohol (standard drinks) purchased per capita aged 12+ years (95% CI)	Mean price (A$) paid per standard drink (95% CI)	Mean amount (A$) spent on alcohol per shopping trip (95% CI)	Mean volume of alcohol (standard drinks) purchased per shopping trip (95% CI)	Mean annual number of shopping trips to purchase alcohol (95% CI)
5th quintile (20% heaviest consumers)	28 (3.16)	2,808.59 (2,719.06–2,898.12)	1.31 (1.20–1.41)	67.15 (64.24–70.04)	73.93 (70.88–76.97)	78.22 (76.80–79.63)
4th quintile	52 (5.88)	853.08 (844.76–861.40)	1.18 (1.12–1.24)	51.87 (49.94–53.79)	56.97 (55.38–58.55)	39.99 (39.19–40.79)
3rd quintile	91 (10.28)	361.79 (358.35–365.21)	1.72 (1.63–1.80)	55.42 (53.78–57.04)	45.34 (43.98–46.68)	23.21 (22.72–23.68)
2nd quintile	147 (16.61)	169.74 (168.19–178.21)	1.91 (1.81–1.99)	49.13 (47.81–50.43)	36.19 (35.46–36.91)	15.54 (15.12–15.94)
1st quintile (20% lightest consumers)	567 (64.07)	47.67 (46.54–48.80)	2.21 (2.10–2.31)	37.60 (36.51–38.67)	24.39 (23.73–25.04)	6.46 (6.2–6.71)
All Households	885 (100.00)	834.4 (809.58–859.26)	1.66 (1.62–1.70)	52.14 (51.29–52.99)	47.20 (46.39–48.01)	32.37 (31.81–32.93)

^a^ Consumption volume is the volume of pure alcohol purchased by each of the five quintiles of households, where each quintile represents 20% of the cumulative volume of alcohol purchased by the entire sample.

CI refers to Confidence Interval.

The question of substitution between alcoholic products when prices change is also important for assessing all possible ramifications of pricing and taxation changes. However, there is currently insufficient evidence available on cross-price elasticities for Australia that could be applied to our study and have a significant effect on the results. There is also a scarcity of such evidence more generally at the international level and further research is warranted in this area, as Babor *et al* [19] notes there have been no systematic reviews of cross-price elasticities between alcohol beverage categories.

Among the few studies internationally that report cross-price elasticities, these generally find very low substitution between alcohol product categories. Modelling by Purshouse *et al* [30] report separate cross-price elasticities for moderate and hazardous/harmful drinkers in England, differentiated by on-trade and off-trade beer, wine, spirits, and RTDs (i.e. 8×8 product categories). The cross-price elasticities they report for off-trade alcohol, which includes substitution from off-trade beverages to other off-trade beverages and to the on-trade equivalent beverage, are of a very low magnitude for moderate drinkers (less than 0.01% change in consumption following a 1.0% price increase) and also very low for hazardous/harmful drinkers (less than 0.05% change in consumption following a 1.0% price increase). More recent estimates of cross-price elasticities for the UK report similar findings: a mix of positive and negative signs numbering 46 and 44 respectively and only 6 out of 90 were statistically significant, among which 5 out of 6 have positive signs [64]. Australian modelling by Fogarty [65] reports cross-price elasticties, but only between spirits and RTDs, and does not differentiate by on-trade or off-trade. Like the international studies, the cross-price elasticities for spirits/RTDs reported by Fogarty are very low (0.06%–0.08% for ‘abusive consumers’).

## Results

### Distribution of alcohol consumption

Table 4 presents the summary descriptive statistics and corresponding confidence intervals for the sample as a whole and by quintile of apparent consumption. We use the command “ci” in Stata to calculate confidence interval. It uses the sample mean, variance and two sided t-stats to calculate lower and upper bounds of confidence interval. For clarity, we do not report alcohol ‘use’ or ‘drinking’ in the discussion below. However, we use the terms ‘purchase’, ‘apparent consumption’, and ‘consumers’ of alcohol inter-changeably.

The mean annual number of standard drinks purchased per capita among all households is 834.4 (95% CI 809.58–859.26). However, the distribution of the alcohol consumption among the sample of households is highly unequal. Those in the 1^st^ quintile (the lightest 20% of consumers) on average purchase 47.7 standard drinks per year (95% CI 46.54–48.80), while those in the 5^th^ quintile (the heaviest 20% of consumers) on average purchase 2808.5 standard drinks per year (95% CI 2719.06–2898.12). Together, households in the 4^th^ and 5^th^ quintile account for only 10% of the total sample, yet consume 40% of the total volume of alcohol, with an average apparent consumption above the recommended level for low-risk of harm over the lifetime (i.e. no more than 2 standard drinks/day over the lifetime). In contrast, the 1^st^ quintile represents 64% of the total sample, yet consume only 20% of the total volume of alcohol.

### Differences in prices paid, purchase frequency and product preferences

The mean price paid per standard drink among all households is $1.66 (range 0.194–13.23; 95% CI 1.62–1.70), but this differs significantly between the heaviest consumers (A$1.31 (95% CI 1.20–1.41)) and the lightest consumers ($2.21 (95% CI 2.10–2.31)). Although the heaviest consumers show a preference for relatively cheaper alcohol, we find that they are by far the highest and most frequent spenders in the sample. Compared to the lightest consumers, the heaviest consumers spend 79% (A$29.55) more on alcohol per shopping trip, purchase three times as much alcohol (49.5 standard drinks) per shopping trip, and make 12 times the number of shopping trips to purchase alcohol per year.

**Figure 1:**
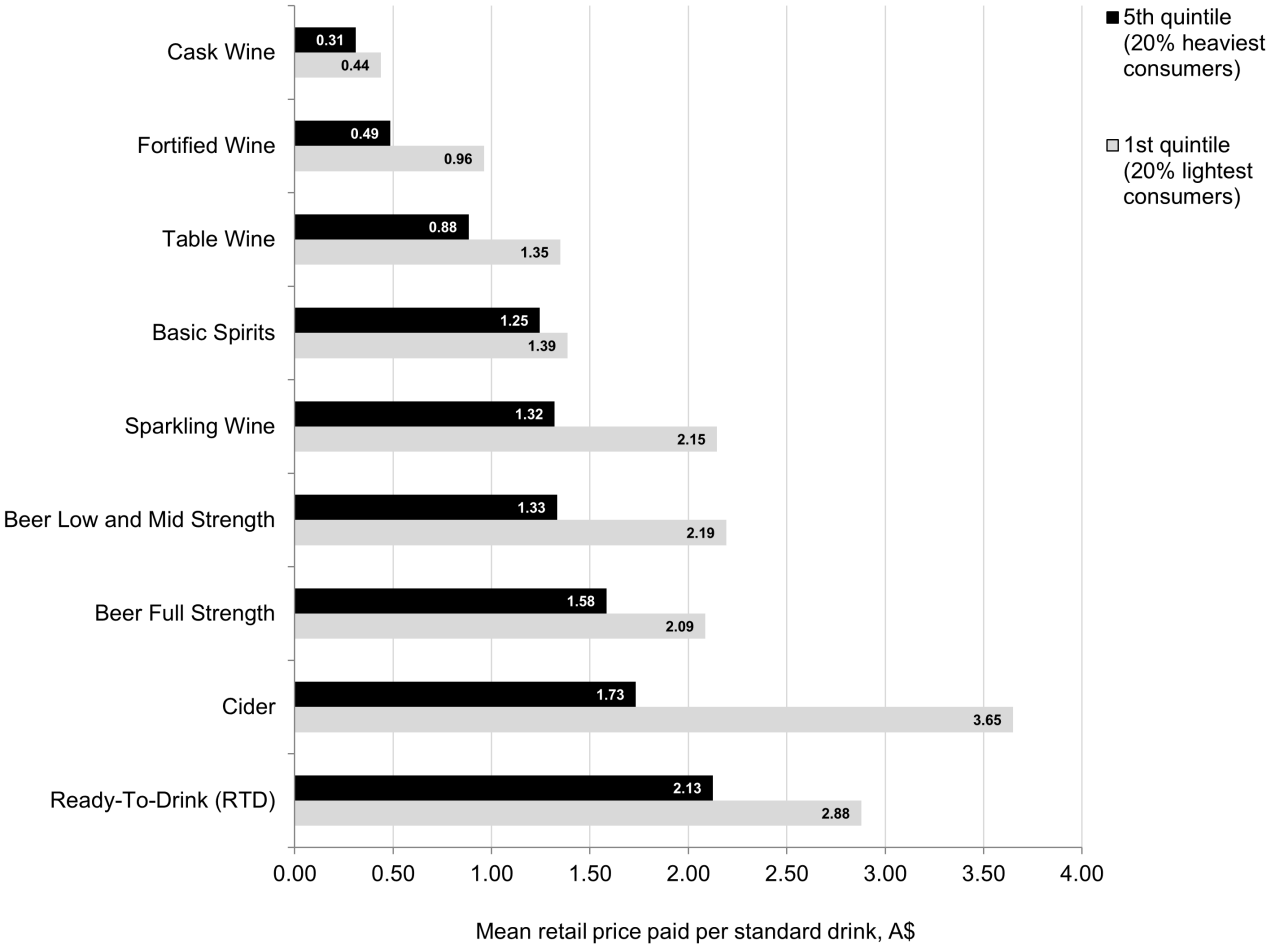
Mean retail price paid (A$) per standard drink for selected alcohol product types, 1^st^ and 5^th^ consumption quintiles.

Among all quintiles, the product type that is purchased in the highest volume is cask wine [see Table 5]. The contribution of cask wine to heavy consumption of alcohol is profound. Consumption of cask wine among the heaviest consumers (4^th^ and 5^th^ quintiles) alone accounts for 83% of the total cask wine consumed by the entire sample of households. This type of alcohol is also the cheapest to purchase, costing $0.34 per standard drink on average (95% CI 0.33–0.35) [see Table 6]. Fortified wine, which has the highest alcohol content of all wine product categories (17.5% ABV on average), is also relatively cheap, costing $0.72 per standard drink on average (95% CI 0.67–0.76). We find that compared to the lightest consumers, the heaviest consumers favour cheaper products, and this is consistent across all product categories [see Figure 1]. With regards to cask wine, the heaviest consumers pay a mean price of only A$0.31 per standard drink (95% CI 0.304–0.316), compared to the lightest consumers who pay a mean price of A$0.44 per standard drink (95% CI 0.418–0.456). The difference in mean price paid between the heaviest and lightest alcohol consumers is even greater with regards to fortified wine ($0.49 compared to $0.96 per standard drink).

**Table 5:** Mean annual per capita volume of alcohol purchased (number of standard drinks) in each product category by quintile volumes.

Apparent consumption volume	Wine – cask (95% CI)	Beer full strength (95% CI)	Wine – fortified (95% CI)	Spirits (95% CI)	Wine – sparkling (95% CI)	Wine – table (95% CI)	Beer mid/low strength (95% CI)	RTDs (95% CI)	Cider (95% CI)
5^th^ quintile (20% heaviest consumers)	3,876.8 (3680–4073)	2,771.5 (2590–2952)	1,013.0 (936–1089)	957.0 (913–1001)	798.7 (714–883)	659.6 (638–680)	655.0 (620–689)	176.6 (159–193)	59.3 (45–72)
4^th^ quintile	1,222.8 (1192–1253)	746.0 (718–773)	283.2 (270–295)	246.3 (237–255)	117.7 (109–125)	270.3 (265–275)	286.9 (281–292)	64.5 (62–66)	24.3 (21–27)
3^rd^ quintile	635.4 (621–648)	294.7 (287–301)	126.0 (121–130)	80.2 (77–82)	34.1 (32–35)	121.3 (118–123)	93.0 (86–99)	29.7 (28–30)	12.2 (10.6–13.6)
2^nd^ quintile	294.2 (287–301)	108.3 (105–111)	51.4 (48–54)	35.0 (33–36)	15.0 (14.4–15.5)	53.7 (52–54)	29.1 (26–32)	15.0 (14.4–15.5)	3.9 (3.3–4.4)
1^st^ quintile (20% lightest consumers)	100.1 (94–105)	20.8 (19–22)	9.5 (08–10)	9.2 (8.6–9.7)	4.9 (4.6–5.2)	13.8 (13.2–14.4)	5.2 (4.5–5.8)	4.4 (4.1–4.8)	1.5 (1.2–1.7)

**Table 6:** Original mean retail prices paid and estimated new mean retail prices after selected pricing and taxation polices are applied, by product type, A$.

	Original mean retail price paid		New mean after tax retail price per product after a MUP or uniform volumetric tax^a^ is applied				
Product type (container size, number of standard drinks)	Per standard drink	Per product	Minimum price of A$0.50 per standard drink	Minimum price of A$1.00 per standard drink	Minimum price of A$1.50 per standard drink	Minimum price of A$2.00 per standard drink	Volumetric tax of A$41.68 per LAL
Beer Full Strength (375 ml, 1.4)	1.73	2.42	2.42	2.46	2.77	3.26	2.51
Beer Low and Mid strength (375 ml, 1.0)	1.76	1.76	1.76	1.77	1.90	2.20	1.81
Table Wine (750 ml, 8.0)	1.26	10.09	10.25	11.41	13.85	17.11	13.11
Fortified Wine (750 ml, 11.0)	0.72	7.93	8.48	12.53	17.30	22.42	12.95
Cask Wine (4,000 ml, 36.0)	0.34	12.38	18.65	36.00	54.00	72.00	29.75
Sparkling Wine (750 ml, 6.5)	1.92	12.51	12.51	12.69	13.94	16.07	14.41
Ready-To-Drink (RTD) Spirits (375 ml, 1.4)	2.52	3.53	3.53	3.54	3.56	3.65	2.78
Brandy (700 ml, 20.0)	1.38	27.51	27.52	27.59	31.69	40.96	19.08
Basic Spirits (700 ml, 22.0)	1.33	29.29	29.37	29.62	33.85	44.00	19.99
Premium Spirits (700 ml, 22.0)	7.52	165.52	165.52	165.52	165.52	165.66	154.75
Cider (355 ml, 1.4)	2.37	3.32	3.34	3.37	3.43	3.65	2.57

^a^ The first 1.15% of alcohol content in all beverages is exempt from the uniform volumetric tax.

### Effects of a minimum price and new volumetric tax on retail prices

By applying a MUP of A$1.00 per standard drink, we estimate that only cask wine and fortified wine would increase substantially in price [see Table 6]. The estimated mean retail price of a 4-litre cask of wine containing 36 standard drinks would increase by 190% to $36.00, and the estimated mean retail price of a 750 ml bottle of fortified wine containing 11 standard drinks would increase by 58% to $12.53. The mean retail prices of sparkling and table wine, beer, spirits, and RTDs are almost unaffected by a MUP. In contrast, applying a uniform volumetric tax all alcohol products of $41.68 per LAL (with zero tax on the initial 1.15% of alcohol) affects prices of all product types, but in different ways. While the mean retail price of beer and wine products would increase, other products, such as spirits and cider would potentially decrease in price.

### Estimated changes in costs for consumers and effects on purchasing

Changes in the estimated mean annual cost of alcohol purchases per capita for each consumption quintile as a result of introducing a MUP and a new uniform volumetric tax are shown in Table 7.

**Table 7:** Comparison of the effects of a MUP for alcohol and a new uniform volumetric tax on the estimated mean annual change in the cost (A$) and volume (litres of alcohol (LALS)) purchased per capita (persons aged 12+ years in each household) of wine and beer, by quintiles.

		Minimum price for alcohol of A$1.00 per standard drink			Uniform volumetric tax of A$41.68 per litre of alcohol		
		Mean annual change in cost per capita, A$	Mean annual change in volume of alcohol purchased per capita, LALs, %		Mean annual change in cost per capita, A$	Mean annual change in volume of alcohol purchased per capita, LALs, %	
**Wine**	5th quintile (20% heaviest consumers)	393.60	−15.78	−52.23	552.46	−9.56	−36.37
	4th quintile	68.13	−5.17	−53.36	136.01	−3.32	−40.22
	3rd quintile	20.54	−2.27	−52.46	55.91	−1.54	−41.05
	2nd quintile	9.42	−0.79	−40.10	26.79	−0.59	−36.00
	1st quintile (20% lightest consumers)	2.16	−0.13	−26.67	7.12	−0.12	−29.74
**Beer**	5th quintile (20% heaviest consumers)	108.26	−1.85	−8.89	163.92	−0.49	−1.99
	4th quintile	12.54	−0.27	−1.88	42.08	−0.15	−1.97
	3rd quintile	6.58	−0.18	−3.63	17.36	−0.07	−2.44
	2nd quintile	1.17	−0.01	−0.45	5.61	−0.02	−2.08
	1st quintile (20% lightest consumers)	0.14	0.00	−0.48	1.10	0.00	−1.67

We also present the estimated changes to the mean annual volume of alcohol purchased per capita resulting from changes in the cost of alcohol to consumers. Due to the negligible effect of a MUP on consumers of spirits, and for the purposes of brevity and space, we present results for wine and beer consumers only. We find that both a MUP and a new uniform volumetric tax have little effect on changing the overall cost of purchasing wine and beer among light and moderate consumers, and likewise little effect upon the volume of alcohol they purchase. However, with regards to the heaviest consumers, we estimate that a MUP would have a substantial effect on reducing the mean volume of wine (−15.78 LALs) and beer (−1.85 LALs) purchased. While it is less effective than a MUP, a new uniform volumetric tax would still have a large effect on reducing the mean volume of wine and beer purchased (−9.56 and −0.49 LALs, respectively).

Importantly, we estimate that a MUP would result in a much smaller increase in the mean annual per capita cost for the heaviest consumers of wine (A$393.60) and beer (A$108.26), compared to a new uniform volumetric tax on wine and beer (A$552.46 and A$163.82, respectively). That is, our analysis reveals that a MUP would not only achieve greater reductions in heavy consumption than a new uniform volumetric tax, it would also achieve this at a lower cost to consumers.

Further, our analysis shows that the proportion of total cost incurred by light and moderate consumers would rise incrementally as the value of the MUP per standard drink increases: $1.00 = 7%; $1.50 = 16%; $2.00 or more = 21%. This underlines the attractiveness of a MUP of $1.00, as it produces only a small increase in the cost for the lightest consumers but results in significant increases in the cost for the heaviest consumers, and in turn significantly reduces their consumption.

## Sensitivity Analysis

### Effect of tax pass-through rates

Our analysis assumes that a new uniform volumetric tax on alcohol is fully passed through (pass-through rate = 1) to after tax retail prices. This is a reasonable assumption in a perfectly competitive market with constant marginal costs of production. However, in less competitive markets the tax pass-through rate could be less than or greater than 1 depending on the type of market structure. We re-estimate the cost increase to consumers resulting from a new uniform volumetric tax using assumptions of varying tax pass-through rates [see Table 8]. For beer, we compare estimates derived from pass-through rates reported by Kenkel [54] for off-premise beer (minimum: 0.87; full: 1.0; and, average: 1.67). Similarly, for wine we compare estimates derived from three pass-through rates (0.5, 1 and 1.5) that reflect low, full, and high pass through rates, as generally found in the literature. We find that there is no significant cost increase for light and moderate consumers of beer or wine resulting from any of the three different tax pass-through rates tested, and that it is mostly heavy consumers that are affected by a new uniform volumetric tax on alcohol, irrespective of the tax pass-through rates assumed.

**Table 8:** Comparison of different pass through rates on the effects of a new uniform volumetric tax on the estimated mean annual change in the cost (A$) and volume (litres of alcohol (LALs)) purchased per capita (persons aged 12+ years in each household) of wine and beer, by quintiles.

		Mean annual change in cost per capita, A$			Mean annual change in volume of alcohol purchased per capita, LALS			Mean annual change in volume of alcohol purchased per capita, %		
**Wine**	**Pass-through rate**	**0.5**	**1.0**	**1.5**	**0.5**	**1.0**	**1.5**	**0.5**	**1.0**	**1.5**
	5th quintile (20% heaviest consumers)	348.74	552.46	335.33	4.40	9.56	19.87	16.54	36.37	76.03
	4th quintile	90.15	136.01	115.30	1.48	3.32	6.99	17.71	40.22	85.39
	3rd quintile	29.16	55.91	71.80	0.67	1.54	3.28	17.60	41.05	88.26
	2nd quintile	13.43	26.79	33.54	0.25	0.59	1.26	14.99	36.00	78.21
	1st quintile (20% lightest consumers)	2.82	7.12	10.26	0.05	0.12	0.26	11.96	29.74	65.67
**Beer**	**Pass-through rate**	**0.87**	**1.0**	**1.67**	**0.87**	**1.0**	**1.67**	**0.87**	**1.0**	**1.67**
	5th quintile (20% heaviest consumers)	13.83	163.92	871.32	0.041	0.49	2.79	0.165	1.99	11.39
	4th quintile	3.57	42.08	216.45	0.012	0.15	0.84	0.164	1.97	11.26
	3rd quintile	1.48	17.36	87.12	0.006	0.07	0.41	0.203	2.44	13.98
	2nd quintile	0.48	5.61	28.48	0.002	0.02	0.12	0.173	2.08	11.90
	1st quintile (20% lightest consumers)	0.09	1.10	5.72	0.000	0.00	0.02	0.139	1.67	9.59

### Effect of applying a MUP and a new uniform volumetric tax simultaneously

As discussed earlier, in the context of current policy discussion in Australia, volumetric tax and a MUP are framed as two alternative options. However, it may be feasible for government to implement combinations of the two, and it may also be desirable to do so given that a volumetric tax will not necessarily prevent some alcohol from being sold below cost or given away. In this way, the two policies would potentially complement each other if introduced simultaneously. We estimate the difference in cost increases for consumers if both a new uniform volumetric tax of $41.68 per LAL and MUP of $1.00 per standard drink were applied to wine products simultaneously [see Table 9]. We find that compared with applying a new uniform volumetric tax alone, there would be only small changes in the cost of alcohol for consumers in all quintiles if both a MUP and new volumetric tax were applied simultaneously. Likewise, there would be only small differences in the change in consumption among light, moderate and heavy consumers resulting from applying both policies simultaneously compared with applying a new uniform volumetric tax only. With regards to beer, after a new uniform volumetric tax of $41.68 per LAL is applied, we estimate that no beer products would have after tax retail prices below $1.00 per standard drink, and, thus applying MUP of $1.00 per standard drink in this situation appears to be redundant.

**Table 9:** Estimated effects of applying both a MUP for alcohol and a new uniform volumetric tax simultaneously compared with applying a new uniform volumetric tax alone.

		Applying both a new uniform volumetric tax and MUP simultaneously			Applying a new uniform volumetric tax alone		
		Mean annual change in cost per capita, A$	Mean annual change in volume of alcohol purchased per capita, LALS, %		Mean annual change in cost per capita, A$	Mean annual change in volume of alcohol purchased per capita, LALS, %	
**Wine**	5th quintile (20% heaviest consumers)	517.63	−11.92	−44.64	552.46	−9.56	−36.37
	4th quintile	120.53	−3.93	−47.00	136.01	−3.32	−40.22
	3rd quintile	53.29	−1.86	−49.60	55.91	−1.54	−41.05
	2nd quintile	26.03	−0.70	−41.80	26.79	−0.59	−36.01
	1st quintile (20% lightest consumers)	6.97	−0.14	−33.96	7.12	−0.12	−29.74

### Effects of household size and composition

As discussed earlier, while we assume that all adults in a household consume the same volume of alcohol and that elasticity is constant across household members, this may not be the case. Therefore, we test the robustness of our methodology by comparing household quintiles constructed according to two different methods: (A) assumes all alcohol purchased is consumed equally among all adults in the household; and, (B) assumes all alcohol purchased is consumed by only one person in the household [see Table 10]. We find that using either method A or B makes little difference to which consumption quintiles (heavy, moderate, or light consumers) the households in our sample are allocated. For 92% of households, the quintile to which they were allocated using method A remains the same when method B is applied. Furthermore, given the low magnitude of elasticities (ranging from −0.26 to −0.53), any bias introduced due to our assumption of constant elasticity will be minimal.

**Table 10:** Distribution of household types by level of consumption.

				4th and 5th quintile (heavy purchasers), number of households		1st quintile (light purchasers), number of households	
Household Type	Number of households	% of total	Persons in household who are assumed to be consumers	Equal consumption among adults (Method A)	Only one adult consumes (Method B)	Equal consumption among adults (Method A)	Only one adult consumes (Method B)
Singles and Couples	361	40.79	Adults only	49	44	215	215
Adult Households (age 45+ years)	227	25.65	Adults only	7	10	151	143
Young Families (all children aged <11 years)	133	15.03	Adults only	12	12	119	119
Older families (all children aged >11 years)	164	18.53	Adults and children	12	12	107	105
All Households	885	100.00		80	78	592	582

We also consider the effect of household composition upon our estimates of alcohol consumption, including whether the level of consumption by households comprising large families with teenage children could bias our estimates. The average household size in our sample is relatively small (2.1 adults per household), with 23% of households comprising only one adult and 54% comprising two adults. In terms of ‘life stage’ categories of households (which are the demographic descriptors supplied to us by Nielsen with the HomeScan dataset), 65% of households are categorised as *singles*, *couples* or *adult* households, and 15% are categorised as *young families with all children less than 11 years*. Only 18% of households are categorised as *older families where all children are more than 11 years*, where there is some probability of alcohol consumption by a non-adult (person aged between 12 and 19 years old). Using these classifications of household types, we examine whether the distribution of different household types across the quintiles differs when applying method A or method B to estimate consumption. We find that the allocation of households to the light, moderate or heavy quintiles remains unchanged for most of the sample, irrespective of using method A or method B to estimate consumption levels.

## Discussion

The findings of our study contribute to understanding alcohol purchasing behaviour across the population and have implications for alcohol pricing and taxation policy. While empirical studies in Canada show the effect of minimum prices on overall consumption [26], [27], and modelling studies in the UK have compared the estimated effect of various minimum price thresholds, restrictions on discounted price-promotions, and percentage increases in the retail price of alcohol [20], [29], [30], the advantage of our study is that it compares the estimated effects of a MUP and a volumetric tax according to different levels of consumption, and uses highly detailed electronically-scanned records of household-level alcohol expenditure for this analysis.

Our analysis indicates that a MUP would impact most on those who consume high volumes of alcohol. We find a new uniform volumetric tax would achieve somewhat less reductions in heavy consumption than a MUP, and would result in relatively greater increases in the cost for light, moderate and heavy consumers. However, we do not conclude that a new uniform volumetric tax is an inferior policy option to a MUP, nor do we wish to suggest that they are mutually exclusive. On the contrary, both policies could potentially be complementary, particularly as much of a MUP's usefulness appears to lie in addressing the failings of the current *ad valorem* tax arrangements for wine. We find that applying both a MUP and a new uniform volumetric tax simultaneously would not adversely affect light and moderate drinkers any more than applying a new uniform volumetric tax alone, and together would still have the desired effect of reducing heavy consumption. Our study clearly shows the conspicuous role that cheap wine plays in heavy consumption of alcohol, and that either of the two pricing policy options, or combined, could be effective in significantly changing consumption behaviour.

Some commentators have suggested that a drawback of a MUP in many jurisdictions is that the increased revenue resulting from inflating the price of some products would potentially remain with privately licensed retailers of alcohol [66], and would hence be foregone by government unless a means of recouping it were implemented. The appeal of a new uniform volumetric tax, on the other hand, is that most of the increased revenue is collected by government through well-established mechanisms, and therefore returned to the community. However, careful consideration of the optimal rate (or rates) of a new volumetric tax to effectively discourage heavy consumption will be critical.

Overall, our findings underline the importance of either a MUP or a volumetric tax for a public health orientated alcohol taxation system. We have not considered the complementary effects of other alcohol polices that could be implemented concurrently with a MUP or volumetric taxation, but these remain very important. Controls on the physical availability of alcohol, strong enforcement of liquor laws, restrictions on advertising, health service interventions, and public education campaigns may also play a key role in reducing the alcohol disease burden, and the impact of a MUP and volumetric tax reform would potentially be greater if implemented as part of a multi-pronged alcohol strategy.

A limitation of our study is the lack of matched records of households' drinking patterns with alcohol purchases in our dataset, under-reporting of some off-trade alcohol purchases, the lack of records for on-trade purchases, and not accounting for possible substitution between products, although this is unlikely given a MUP minimises the availability of cheap alternatives.
